# Repertory of Characteristics

**Published:** 1888-02

**Authors:** 


### THE REPERTORY OF CHARACTERISTICS.

So many inquiries are sent us concerning the publication of this repertory that we feel an explanation is necessary. The work on a repertory is one that needs truth and reliability to make it of use. Though a repertory should be used simply as an *index* to the *Materia Medica*, not as a book for prescribing upon, still it must needs be accurate and reliable to be at all serviceable.

When this repertory was first advertised, its publication was promised immediately, and such was our expectation at that time. But when we commenced to edit our notes and the various MSS. received from Dr. C. Lippe and others, we found so many contradictions, so much confusion, and so many errors, that a complete revision of the entire work by a thorough and systematic comparison of the materia medica was considered necessary. This revision is now well under way, and, when done, the result will, we feel confident, more than justify the delay, and fully compensate for the labor expended.

The result will be (we hope) a more complete and more reliable repertory than ever before published. Is not this worth the delay and the labor?

Had not sickness delayed the editor in his work, this revision would have been completed some time ago. The repertory will be published as soon as is possible consistent with faithful work; it will be entirely new, fresh from the materia medica, and we trust all will find it of service in their daily labors.

